# Supplementary material for: HIVprotI: an integrated web based platform for prediction and design of HIV proteins inhibitors
Source: J Cheminform. 2018 Mar 9;10:12. doi: 10.1186/s13321-018-0266-y (PMC5845081; doi:10.1186/s13321-018-0266-y)
Supplement: Supplementary file 2 — Additional file 2. Source code of HIVProtI web server. [file 13321_2018_266_MOESM2_ESM.zip › HIVprotI_Source-code/hivprotI/weka-3-6-6/doc/weka/classifiers/functions/supportVector/Kernel.html]

Kernel


---


|  |  |  |
| --- | --- | --- |
| **Overview** \| **Package** \| **Class** \| **Tree** \| **Deprecated** \| **Index** \| **Help** \| **Weka's home** | |  |
| **PREV CLASS**   **NEXT CLASS** | **FRAMES**    **NO FRAMES**     **All Classes** |  |
| SUMMARY: NESTED \| FIELD \| CONSTR \| METHOD | DETAIL: FIELD \| CONSTR \| METHOD |  |


---


## weka.classifiers.functions.supportVector Class Kernel

```
java.lang.Object
  weka.classifiers.functions.supportVector.Kernel
```

**All Implemented Interfaces:**: java.io.Serializable, CapabilitiesHandler, OptionHandler, RevisionHandler

**Direct Known Subclasses:**: CachedKernel, PrecomputedKernelMatrixKernel, StringKernel

---

``` public abstract class Kernel extends java.lang.Object implements java.io.Serializable, OptionHandler, CapabilitiesHandler, RevisionHandler ```

Abstract kernel.
Kernels implementing this class must respect Mercer's condition in order
to ensure a correct behaviour of SMOreg.

**Version:**
:   $Revision: 5517 $

**Author:**
:   Eibe Frank (eibe@cs.waikato.ac.nz), FracPete (fracpete at waikato dot ac dot nz)

**See Also:**: Serialized Form

---

**Constructor Summary**

`Kernel()`


| **Method Summary** | |
| --- | --- |
| `void` | `buildKernel(Instances data)`             builds the kernel with the given data |
| `java.lang.String` | `checksTurnedOffTipText()`             Returns the tip text for this property |
| `abstract  void` | `clean()`             Frees the memory used by the kernel. |
| `java.lang.String` | `debugTipText()`             Returns the tip text for this property |
| `abstract  double` | `eval(int id1, int id2, Instance inst1)`             Computes the result of the kernel function for two instances. |
| `static Kernel` | `forName(java.lang.String kernelName, java.lang.String[] options)`             Creates a new instance of a kernel given it's class name and (optional) arguments to pass to it's setOptions method. |
| `Capabilities` | `getCapabilities()`             Returns the Capabilities of this kernel. |
| `boolean` | `getChecksTurnedOff()`             Returns whether the checks are turned off or not. |
| `boolean` | `getDebug()`             Gets whether debugging output is turned on or not. |
| `java.lang.String[]` | `getOptions()`             Gets the current settings of the Kernel. |
| `java.lang.String` | `getRevision()`             Returns the revision string. |
| `abstract  java.lang.String` | `globalInfo()`             Returns a string describing the kernel |
| `java.util.Enumeration` | `listOptions()`             Returns an enumeration describing the available options. |
| `static Kernel[]` | `makeCopies(Kernel model, int num)`             Creates a given number of deep copies of the given kernel using serialization. |
| `static Kernel` | `makeCopy(Kernel kernel)`             Creates a deep copy of the given kernel using serialization. |
| `abstract  int` | `numCacheHits()`             Returns the number of dot product cache hits. |
| `abstract  int` | `numEvals()`             Returns the number of kernel evaluation performed. |
| `void` | `setChecksTurnedOff(boolean value)`             Disables or enables the checks (which could be time-consuming). |
| `void` | `setDebug(boolean value)`             Enables or disables the output of debug information (if the derived kernel supports that) |
| `void` | `setOptions(java.lang.String[] options)`             Parses a given list of options. |

**Methods inherited from class java.lang.Object**

`equals, getClass, hashCode, notify, notifyAll, toString, wait, wait, wait`

**Constructor Detail**

### Kernel

```
public Kernel()
```


**Method Detail**

### globalInfo

```
public abstract java.lang.String globalInfo()
```

:   Returns a string describing the kernel

    :   **Returns:**: a description suitable for displaying in the explorer/experimenter gui

---


### eval

```
public abstract double eval(int id1,
                            int id2,
                            Instance inst1)
                     throws java.lang.Exception
```

:   Computes the result of the kernel function for two instances.
    If id1 == -1, eval use inst1 instead of an instance in the dataset.

    :   **Parameters:**: `id1` - the index of the first instance in the dataset: `id2` - the index of the second instance in the dataset: `inst1` - the instance corresponding to id1 (used if id1 == -1) **Returns:**: the result of the kernel function **Throws:**: `java.lang.Exception` - if something goes wrong

---


### clean

```
public abstract void clean()
```

:   Frees the memory used by the kernel.
    (Useful with kernels which use cache.)
    This function is called when the training is done.
    i.e. after that, eval will be called with id1 == -1.

---


### numEvals

```
public abstract int numEvals()
```

:   Returns the number of kernel evaluation performed.

    :   **Returns:**: the number of kernel evaluation performed.

---


### numCacheHits

```
public abstract int numCacheHits()
```

:   Returns the number of dot product cache hits.

    :   **Returns:**: the number of dot product cache hits, or -1 if not supported by this kernel.

---


### listOptions

```
public java.util.Enumeration listOptions()
```

:   Returns an enumeration describing the available options.

    :   **Specified by:**: `listOptions` in interface `OptionHandler`
    :   **Returns:**: an enumeration of all the available options.

---


### setOptions

```
public void setOptions(java.lang.String[] options)
                throws java.lang.Exception
```

:   Parses a given list of options.

    :   **Specified by:**: `setOptions` in interface `OptionHandler`
    :   **Parameters:**: `options` - the list of options as an array of strings **Throws:**: `java.lang.Exception` - if an option is not supported

---


### getOptions

```
public java.lang.String[] getOptions()
```

:   Gets the current settings of the Kernel.

    :   **Specified by:**: `getOptions` in interface `OptionHandler`
    :   **Returns:**: an array of strings suitable for passing to setOptions

---


### setDebug

```
public void setDebug(boolean value)
```

:   Enables or disables the output of debug information (if the derived
    kernel supports that)

    :   **Parameters:**: `value` - whether to output debugging information

---


### getDebug

```
public boolean getDebug()
```

:   Gets whether debugging output is turned on or not.

    :   **Returns:**: true if debugging output is produced.

---


### debugTipText

```
public java.lang.String debugTipText()
```

:   Returns the tip text for this property

    :   **Returns:**: tip text for this property suitable for displaying in the explorer/experimenter gui

---


### setChecksTurnedOff

```
public void setChecksTurnedOff(boolean value)
```

:   Disables or enables the checks (which could be time-consuming). Use with
    caution!

    :   **Parameters:**: `value` - if true turns off all checks

---


### getChecksTurnedOff

```
public boolean getChecksTurnedOff()
```

:   Returns whether the checks are turned off or not.

    :   **Returns:**: true if the checks are turned off

---


### checksTurnedOffTipText

```
public java.lang.String checksTurnedOffTipText()
```

:   Returns the tip text for this property

    :   **Returns:**: tip text for this property suitable for displaying in the explorer/experimenter gui

---


### getCapabilities

```
public Capabilities getCapabilities()
```

:   Returns the Capabilities of this kernel. Derived kernels have to
    override this method to enable capabilities.

    :   **Specified by:**: `getCapabilities` in interface `CapabilitiesHandler`
    :   **Returns:**: the capabilities of this object **See Also:**: `Capabilities`

---


### getRevision

```
public java.lang.String getRevision()
```

:   Returns the revision string.

    :   **Specified by:**: `getRevision` in interface `RevisionHandler`
    :   **Returns:**: the revision

---


### buildKernel

```
public void buildKernel(Instances data)
                 throws java.lang.Exception
```

:   builds the kernel with the given data

    :   **Parameters:**: `data` - the data to base the kernel on **Throws:**: `java.lang.Exception` - if something goes wrong

---


### makeCopy

```
public static Kernel makeCopy(Kernel kernel)
                       throws java.lang.Exception
```

:   Creates a deep copy of the given kernel using serialization.

    :   **Parameters:**: `kernel` - the kernel to copy **Returns:**: a deep copy of the kernel **Throws:**: `java.lang.Exception` - if an error occurs

---


### makeCopies

```
public static Kernel[] makeCopies(Kernel model,
                                  int num)
                           throws java.lang.Exception
```

:   Creates a given number of deep copies of the given kernel using
    serialization.

    :   **Parameters:**: `model` - the kernel to copy: `num` - the number of kernel copies to create. **Returns:**: an array of kernels. **Throws:**: `java.lang.Exception` - if an error occurs

---


### forName

```
public static Kernel forName(java.lang.String kernelName,
                             java.lang.String[] options)
                      throws java.lang.Exception
```

:   Creates a new instance of a kernel given it's class name and
    (optional) arguments to pass to it's setOptions method.

    :   **Parameters:**: `kernelName` - the fully qualified class name of the classifier: `options` - an array of options suitable for passing to setOptions. May be null. **Returns:**: the newly created classifier, ready for use. **Throws:**: `java.lang.Exception` - if the classifier name is invalid, or the options supplied are not acceptable to the classifier


---


|  |  |  |
| --- | --- | --- |
| **Overview** \| **Package** \| **Class** \| **Tree** \| **Deprecated** \| **Index** \| **Help** \| **Weka's home** | |  |
| **PREV CLASS**   **NEXT CLASS** | **FRAMES**    **NO FRAMES**     **All Classes** |  |
| SUMMARY: NESTED \| FIELD \| CONSTR \| METHOD | DETAIL: FIELD \| CONSTR \| METHOD |  |


---
